# Annual Meeting of the Union Medical Association, at Richview, Washington Co., Ill.

**Published:** 1857-08

**Authors:** Thos. Wilkins, J. S. Murphy

**Affiliations:** President; Secretary


*Proceedings of the Annual Meeting of the Union Medical Association, held at Richview, Washington County, Ill., May 19th*, 1857.

Pursuant to adjournment, the "Union Medical Association" met in Odd Fellows' Hall, at Richview, May 19th, 1857.

G. W. Hotchkiss, M. D., the former President, took the chair, and called the house to order; when, on motion, J. S. Murphy, M. D., was chosen Secretary *pro tem.*, the former Secretary being absent.

In consequence of the Constitution and former proceedings of the Association being lost, it was moved by Dr. Haller that a committee, consisting of a member from each county, be appointed to draft and report a new Constitution and By-Laws for its future government; whereupon the chair named the following gentlemen as said committee, viz.: B. F. Haller, of Fayette; D. N. Moore, of Clinton; D. H. McCord, of Marion; P. B. Marshall, of Washington; and S. H. Bundy, of Perry. The committee retired to discharge their duty.

On motion of Dr. Dunning, a committee was appointed to prepare and report a Fee Bill for the government of the members of the Association in their charges for professional services. Drs. Dunning, Stearns, Lucas and Barber were named as said committee.

A note was read from Dr. Knapp, regretting his inability to attend the meeting, and expressing a deep interest in the cause.

During the absence of these committees, the remaining members engaged in the discussion of various subjects of interest.

The committee appointed to draft a Constitution and By-Laws presented their report, which was read, and unanimously adopted, and the committee discharged.

On motion, it was ordered that the members should repair to the Secretary's desk, and subscribe their names to the Constitution, which was accordingly done.

The Association then proceeded to elect the regular officers for the ensuing year; whereupon Thomas Wilkins, M. D., was elected President; D. H. McCord, M. D., Vice President; John S. Murphy, M. D., Recording Secretary; S. H. Bundy, M. D., Corresponding Secretary; and B. H. Lucas, M. D., Treasurer.

On taking the chair, the President called for the reports of the standing committees.

Committee on Practice of Medicine—No report.

Committee on Surgery—No report ready.

Committee on Obstetrics—No report.

Committee on Drugs and Medicines—No report.

The proceedings having been lost, it was not certainly known who the chairmen of all the committees were. Dr. Stearns, chairman of the Committee on Surgery, stated that he had been unable to gather from the physicians in Southern Illinois sufficient materials for such a report as he had desired to present, but he had collected some facts, and asked to be continued on the committee, which was granted.

Members appointed at the previous meeting were called upon for their essays, upon which Dr. Moore read an able paper upon Milk Sickness, which gave rise to an animated and interesting discussion touching the cause, symptoms, pathology and treatment of the disease. Drs. Wilkins, Stearns and Barber denied the specific existence of such a disease as is supposed by some to be conveyed into the human system by using the flesh, milk or butter of the cow, and claimed that the symptoms and treatment said to be most successful indicated nothing more than congestion of the stomach and bowels.

Drs. Hotchkiss, Haller, McCord, Moore, Bundy and others maintained that it was a disease *sui generis*, confined to certain localities, more common in the fall season, first attacking cattle, sheep, etc., and thence transferred into the systems of those who use their flesh, milk or butter; having its origin, as they supposed, in some unknown specific poison.

After a general interchange of views, it was agreed to hold the next meeting of the Association in Anna (or Jonesboro), Union County, Ill., commencing on the second Tuesday in November, 1857, with the hope of meeting a large number of the profession in the southern counties.

Standing Committees to report at next meeting were then appointed, as follows:

Committee on Arrangements—Drs. S. S. Condon, J. V. Brooks and H. C. Hacker.

Practical Medicine—Drs. G. W. Hotchkiss, of Nashville (chairman), D. H. McCord and Wm. H. Burns.

Surgery—Drs. A. D. Stearns, of Vandalia (chairman), P. B. Marshall and John W. Cameron.

Obstetrics—Drs. H. B. Lucas, of Richview (chairman), C. W. Dunning and Wm. White.

Drugs and Medicines—Drs. F. B. Haller, of Vandalia (chairman), W. C. Pace and D. N. Moore.

The President then appointed A. D. Stearns, H. Barber and D. N. Moore Censors for the ensuing year.

The committee appointed to draft a Fee Bill presented their report, which, after considerable discussion and a slight amendment, was unanimously adopted, and recommended to the profession in this portion of the State.

On motion, the meeting adjourned one hour for supper, to meet at the church at 7 o'clock, to hear the annual address, and come to order again at the call of the Chair.

At 7 o'clock the Association and citizens of Richview assembled at the church, and listened to the very able address of Dr. F. B. Haller, on the "History of Ancient Medicine."

The address was well delivered, and, for professional learning and historical research, will prove a valuable addition to any library. After the address, the members repaired to "Odd Fellows' Hall," and were again called to order.

On motion of Dr. Hotchkiss,

*Resolved*, That the thanks of the Association are hereby tendered to Dr. Haller, in consideration of his very able address, and he is requested to furnish a copy for publication.

The following gentlemen were appointed delegates to the

State Medical Association, to meet in Chicago, on the first Tuesday in June, 1857, viz.; F. B. Haller, S. S. Bundy, C. W. Dunning and H. B. Lucas.

On motion of Dr. Bundy, S. S. Condon, M. D., of Jonesboro, was elected a member of the Association, and Drs. S. S. Condon, D. H. McCord and James Phillips added to the delegation.

On motion of Dr. Stearns, G. L. Jackson, M. D., was elected a member of the Association.

In compliance with the Constitution, the President appointed G. W. Hotchkiss, M. D., to deliver a public address at the semi-annual meeting in November; H. Barber, M. D., to write an essay on some medical subject; and S. H. Bundy, M. D., to deliver the next annual address.

Dr. A. D. Stearns was appointed to prepare an essay for the next meeting on the use of Quinine in the treatment of Pneumonia and kindred diseases, and Dr. John S. Murphy to prepare an essay on the Causes, Pathology and Treatment of Typhoid Fever.

On motion, the Committee on Publications was directed to print 500 copies of the Constitution and By-Laws, 300 of the Fee Bill, and 500 of the proceedings of this meeting, for distribution in Southern Illinois.

On motion of Dr. Haller,

*Resolved*, That the proceedings of this meeting be published in the "North-Western Medical and Surgical Journal," and all the papers in Southern Illinois.

Dr. Bundy was appointed to take charge of the publication and distribution of the above documents.

It was ordered by the Association that the members report to Dr. Bundy the addresses of the physicians in their respective counties; and that he adopt such measures as will enable him to learn the address of each regular physician in Southern Illinois.

On motion, the Association adjourned to meet in Anna (or Jonesboro), at 11 o'clock A. M., on the second Tuesday in November, 1857.

THOS. WILKINS, M. D., *President*.

J. S. Murphy, M. D., *Secretary*.

